# Predicting a Positive Response to Intravenous Immunoglobulin in Isolated Lower Motor Neuron Syndromes

**DOI:** 10.1371/journal.pone.0027041

**Published:** 2011-10-31

**Authors:** James R. Burrell, Con Yiannikas, Dominic Rowe, Matthew C. Kiernan

**Affiliations:** 1 Neuroscience Research Australia, Sydney, New South Wales, Australia; 2 University of New South Wales, Sydney, New South Wales, Australia; 3 Concord Repatriation General Hospital, Sydney, New South Wales, Australia; 4 Australian School of Advanced Medicine, Macquarie University, Sydney, New South Wales, Australia; National Institute of Health, United States of America

## Abstract

**Objective:**

To determine clinically related characteristics in patients with pure lower motor neuron (LMN) syndromes, not fulfilling accepted diagnostic criteria, who were likely to respond to intravenous immunoglobulin (IVIg) treatment.

**Methods:**

Demographic, clinical, laboratory and neurophysiological characteristics were prospectively collected from patients with undifferentiated isolated LMN syndromes who were then treated with IVIg. Patients were classified as either responders or non-responders to therapy with IVIg based on clinical data and the two groups were compared.

**Results:**

From a total cohort of 42 patients (30 males, 12 females, aged 18-83 years), 31 patients responded to IVIg and 11 did not. Compared to patients that developed progressive neurological decline, responders were typically younger (45.8 compared to 56.0 years, P<0.05) and had upper limb (83.9% compared to 63.6%, NS), unilateral (80.6% compared to 45.5%, P<0.05), and isolated distal (54.1% compared to 9.1%, P<0.05) weakness. Patients with predominantly upper limb, asymmetrical, and distal weakness were more likely to respond to IVIg therapy. Of the patients who responded to treatment, only 12.9% had detectable GM_1_ antibodies and conduction block (not fulfilling diagnostic criteria) was only identified in 22.6%.

**Conclusions:**

More than 70% of patients with pure LMN syndromes from the present series responded to treatment with IVIg therapy, despite a low prevalence of detectable GM_1_ antibodies and conduction block. Patients with isolated LMN presentations, not fulfilling accepted diagnostic criteria, may respond to IVIg therapy, irrespective of the presence of conduction block or GM_1_ antibodies, and should be given an empirical trial of IVIg to determine treatment responsiveness.

## Introduction

From a clinical perspective, it is often difficult to distinguish amyotrophic lateral sclerosis (ALS) from more treatable motor neuropathies early in the course of the illness, particularly in patients with pure lower motor neuron (LMN) involvement. [1] For instance, patients with multifocal motor neuropathy (MMN) also present with lower motor neuron (LMN) syndromes, typically with asymmetrical weakness of the distal upper limbs. Weakness and wasting develop in the absence of objective sensory or upper motor neuron (UMN) dysfunction. The demonstration of focal conduction block (CB) on motor nerve conduction studies remains the key neurophysiological hallmark of MMN, and although anti-ganglioside antibodies (GM1 antibodies) may be detectable in a proportion of patients, such antibodies may also be expressed in ALS. [2]

Although often difficult in clinical practice, the distinction of ALS and other degenerative lower motor neuron diseases from MMN remains crucial as therapy with IVIg is likely to benefit patients with MMN. Specifically, although MMN is rare, up to 78% of patients will improve with intravenous immunoglobulin (IVIg) therapy, whereas patients with ALS will continue to deteriorate. [3], [4] IVIg therapy is expensive and prescription is often restricted by regulatory authorities. In addition to common and mild side effects such as headache, fever, and malaise, therapy with IVIg may occasionally be complicated by nephrotoxicity, [5] anaphylaxis, myocardial infarction, stroke or even death [6] further supporting the general view that IVIg therapy should be reserved for patients likely to benefit. Without treatment, patients with MMN develop progressive weakness and functional disability, and in such a context may be misdiagnosed as ALS. In addition, patients with an MMN-like presentation, but without CB, may also be initially diagnosed as ALS, although a therapeutic treatment trial may show benefit from IVIg. [7]

The current consensus criteria for the diagnosis of MMN rely on the demonstration of CB in two or more motor nerve segments. [8] The criteria were designed for research use rather than clinical practice and inevitably exclude treatable patients from the diagnosis of MMN. Consequently, the present study was prompted by the recognition of a group of patients who presented with an ultimately treatment responsive LMN syndrome, but did not meet the diagnostic criteria. The aim of the present study was to identify clinical and neurophysiological characteristics using a ‘real-life’ practical approach, that may prove useful to predict IVIg response amongst patients, to further dissect patients with a pure LMN syndrome in routine clinical practice.

## Methods

Patients with clinically isolated LMN syndromes were identified from three specialised ALS clinics. Ethical approval for the study was obtained from the South Eastern Sydney and Illawarra Area Health Service Human Research Ethics Committee.

Patients were included in the study if they presented with an undifferentiated isolated LMN syndrome, that did not meet the accepted criteria for either a degenerative motor neuron disease or an inflammatory motor neuropathy (eg MMN or chronic inflammatory demyelinating polyneuropathy) and had received induction treatment with induction IVIg treatment (0.4 g/kg per day for 5 consecutive days) followed by at least three monthly maintenance treatments (0.4 g/kg by single infusion per month). Patients were studied consecutively and data recorded prospectively. Demographic data, symptom duration, presence and pattern of weakness (i.e. distal, proximal or mixed), the presence of wasting, and pattern of onset (unilateral or bilateral, upper limb or lower limb) were all recorded. Therapy was continued until the response to treatment had become clear and, in practice, this often entailed months of maintenance treatment. Treatment was ceased, at the discretion of the treating physician, in patients who deteriorated despite ongoing IVIg treatment, usually due to the development of more typical features of ALS such as UMN signs or bulbar involvement.

The study exclusion criteria were objective sensory deficits or abnormalities on standard sensory nerve conduction studies, marked UMN signs such as pathological hyper-reflexia (defined as exaggerated reflexes elicited with minimal stimulus or spread of reflexes) or an alternative diagnosis. Specifically, patients with chronic inflammatory demyelinating polyneuropathy, or benign focal amyotrophy were excluded from the study. [9], [10] Patients with suspected Hirayama's disease underwent cervical MRI in neck flexion, [11] and if the diagnosis was confirmed, were excluded.

Standard clinical investigations and GM_1_ antibodies were recorded in each patient. Neurophysiological data such as distal compound motor action potential (CMAP) amplitudes and the detection of CB was recorded. Standard neurophysiological investigations were undertaken using an Oxford Teca Synergy machine (Oxford Instruments, Old Woking, Manor Way, UK). Patients had bilateral studies of upper and lower limb nerves including the median, ulnar (with above and below elbow stimulations), common peroneal and tibial motor nerves, with results compared to laboratory normal and published control values. [12] CB was defined in accordance with the consensus criteria for the diagnosis of MMN, [8] such that definite CB was indicated by a reduction in CMAP amplitude of >50% in distal median, distal ulnar or proximal peroneal nerve segments, or >60% in a distal peroneal or tibial nerve segments. CB across common sites of entrapment were not included in the analysis. Probable CB was noted when an amplitude reduction of 40–49% in median and ulnar nerve segments or 50–59% in distal peroneal or tibial nerve segments was detected. In addition, abnormal amplitude reduction (AAR), defined as 30–40% amplitude reduction in median, ulnar and radial nerve segments, or 40–50% amplitude reduction in distal common peroneal and tibial nerve segments was recorded. The detection of electromyographic discharges, fibrillation and fasciculation potentials was also noted. By convention, the frequency of fibrillation potentials recorded in each muscle were graded on a scale from 0 to 4 (0 – None; 1 – persistent fibrillation potentials in at least two areas; 2 – moderate numbers of persistent fibrillation potentials; 3 – large numbers of persistent fibrillation potentials; 4 – profuse, widespread, persistent fibrillation potentials which fill the baseline). [13] The individual muscle grades were summed and divided by the number of muscles studies to determine a novel fibrillation score per muscle for each patient.

Response to treatment was determined clinically through a combination of clinical history and examination findings on follow-up, the latter with reference to any improvement, stabilisation or deterioration in motor power as graded by the medical research council (MRC) grading scales [14] after treatment. Patients who improved or stabilised with IVIg treatment were classified as responders and those who deteriorated in terms of power testing as non-responders. As mentioned, the development of upper motor neuron signs or bulbar dysfunction, or clinical progression suggestive of ALS, was noted and indicated non-response to treatment. Although all patients received a minimum of three months of IVIg therapy, treatment was continued until such time as the clinical outcome had become clear.

Statistical analysis, with P<0.05 considered significant, was performed by application of the chi-square, paired t and Mann-Whitney tests as required (Statistical Package for Social Sciences 17.0, SPSS Inc; Chicago, IL, USA). In order to compare categorical data (for example proximal/mixed weakness compared to distal weakness), 2×2 tables were constructed and the Chi-square test applied. A receiver operator curve (ROC) was constructed using the fibrillation score to plot the true positive rate (y axis) and the false positive rate (x axis) of a candidate investigation. This process was utilised to establish a threshold at which the both the sensitivity and specificity of the investigation were maximal.

## Results

In total, 42 patients were eligible for the study (30 males, 12 females, mean age 48.4 +/- 13.9 years, range 18 to 83 years), and patients were included over a period of 10 years. Mean follow-up duration was 35 +/- 32 months (range 3 to 136 months). After treatment with IVIg, 31 patients were classified as responders; 11 patients as non-responders and later fulfilled the criteria for a diagnosis of ALS. During the study period, two non-responders died of ALS-related complications. All responders continued to receive monthly maintenance IVIg infusions and no significant complications of IVIg treatment were encountered.

### Clinical phenotype

The demographic and clinical characteristics of responders and non-responders are presented in Table 1. The mean age of responders was significantly less (45.8 +/- 13.4 years) than non-responders (56.0 +/- 13.1 years, P<0.05). Median symptom duration prior to diagnosis was longer in responders (18 months) than non-responders (12 months, NS), as was mean symptom duration at first assessment (responders 46.8 +/- 72.3; non-responders 18.2 +/- 20.2, NS). On average, responders had symptoms for 46.8 months prior to treatment, and several responders had symptoms for years before receiving treatment with IVIg.

**Table 1:** Demographic and clinical characteristics.

	Responders	Non-responders	P value
**Number of Patients**	31	11	
**Average Age (years +/- SD)**	45.8 +/-13.4	56.0 +/-13.1	<0.05
**Male Gender (% pts)**	22 (71%)	8 (73%)	NS
**Median Symptom Duration (months)**	18	12	
**Mean Symptom Duration at First Assessment (months +/- SD)**	46.8 +/- 72.3	18.2 +/- 20.2	NS
**Follow-up Duration (months +/- SD)**	41.6 (+/-34.9)	16.9 (+/-9.0)	<0.05
**Number of involved limbs (+/- SD)**	1.5 (+/-0.6)	2.1 (+/-1.3)	NS
**Degree of Wasting**			
None or mild	19 (61.3%)	6 (54.5%)	NS
Marked	12 (38.7%)	5 (45.5%)	
**Pattern of Weakness**			
Upper limb	26 (83.9%)	7 (63.6%)	NS
Unilateral	25 (80.6%)	5 (45.5%)	<0.05
Distal	17 (54.8%)	1 (9.1%)	<0.05

The demographic and clinical features of 42 patients who presented with an isolated LMN syndrome. Responders were younger than non-responders, and typically had distal, asymmetrical, upper limb weakness.

**Figure 1:**
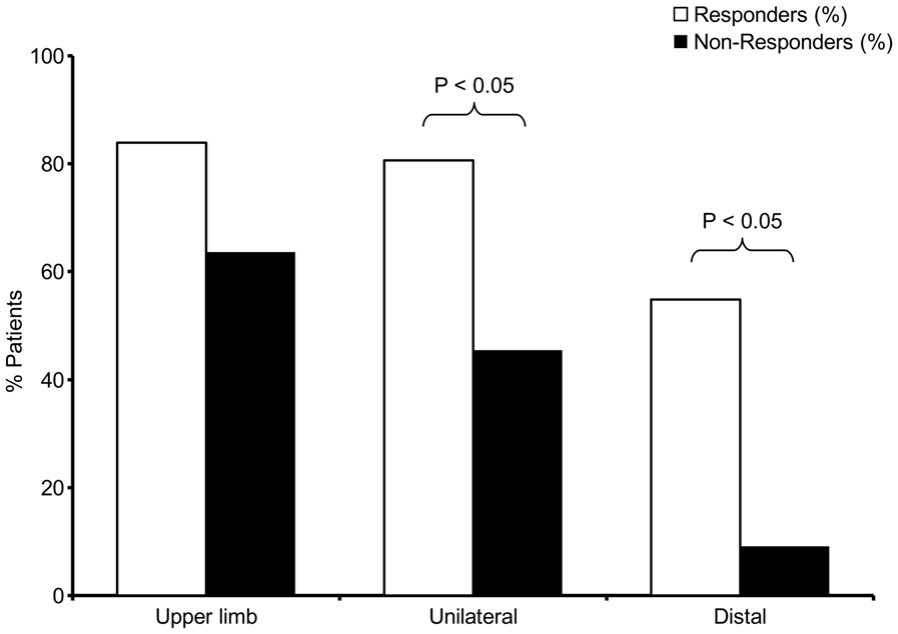
Responders (white bars) had the typical clinical phenotype of MMN, namely upper limb, unilateral, and distal onset weakness.

Weakness was the most prominent presenting feature in both groups, although a minority of patients presented with wasting, muscle cramps or pain. Although severity of muscle wasting did not differ between responders and non-responders, the two patient groups had different patterns of weakness. For example, 54.8% of responders had isolated distal upper limb weakness rather than proximal or mixed weakness, compared to on 9.1% of non-responders (P<0.05). Unilateral onset was more common in responders than non-responders (P<0.05) (Figure 1). Responders also tended to have upper rather than lower limb symptom onset, and had fewer limbs involved at the time of presentation when compared to non-responders. There was no correlation between the degree of limb wasting and treatment outcome.

### Clinical investigations

Results of clinical investigations are summarised in Table 2. GM_1_ (IgM class) antibodies were identified in 12.9% of responders and definite CB (not reaching diagnostic criteria for MMN) was identified in 22.6% of responders, but more than 50% of responders had no evidence of GM_1_ antibodies or CB. When detected, CB was identified in the ulnar nerve (3 patients), the median nerve (2 patients), the common peroneal nerve (1 patient) and the tibial nerve (1 patient). Neither GM_1_ antibodies nor CB was identified in non-responders. Of the 77.4% of responders without definite CB, 6.5% had probable CB and a further 12.9% had ARR, which was also detected in 36.4% of non-responders. In total, 41.9% of responders had CB or ARR compared to 36.4% non-responders, but the difference was not significant. By combining GM_1_ antibodies with CB and ARR to identify responders sensitivity improved (41.9%), but the specificity deteriorated (36.4%).

**Table 2:** Neurophysiological Characteristics.

	Responders	Non-responders	P value
**Number of Patients**	31	11	
**GM_1_ antibodies IgM + (% pts)**	4 (12.9%)	0	NS
**Distal CMAP at initial assessment (mean mV +/- SD)**			
Upper limb	7.3 +/-2.7	8.2 +/-2.3	NS
Lower limb	6.6 +/-3.9	4.3 +/-2.7	NS
Overall	7.0 +/-2.4	6.4 +/-2.2	NS
**Definite CB (% pts)**	22.6%	0.0%	NS
**Definite or Probable CB (% pts)**	29.0%	0.0%	NS
**Definite CB, Probable CB or ARR (% pts)**	41.9%	36.4%	NS
**Electromyography (% pts)**			
Fibrillations	61.3%	81.8%	NS
Complex repetitive discharges	22.6%	36.4%	NS
Fasciculations	45.2%	36.4%	NS

The laboratory and clinical neurophysiological characteristics of 42 patients who presented with an isolated LMN syndrome. GM_1_ antibodies and conduction block (CB), although only identified in responders, were detected in less than half of all responders.

**Figure 2:**
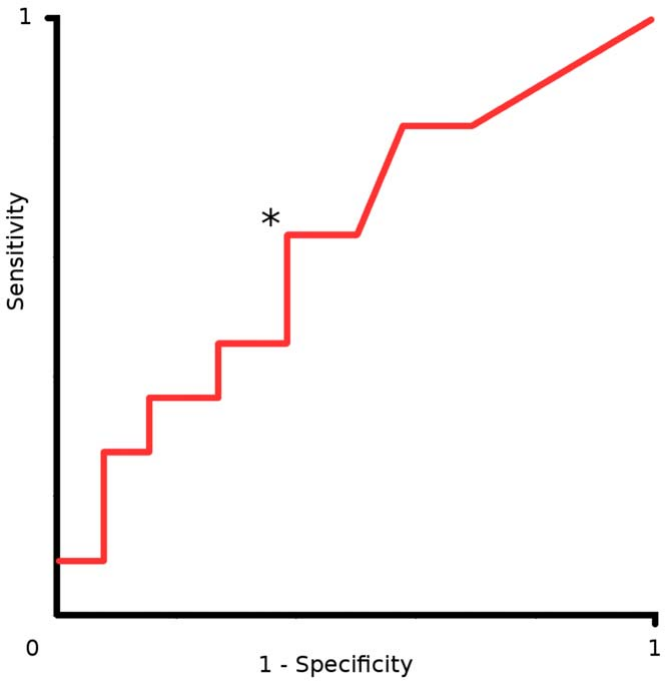
A receiver operator curve (ROC) was constructed to identify the fibrillation score cut-off that identified non-responders with the maximal sensitivity while preserving specificity. Sensitivity is represented on the y axis and (1-specificity) on the x axis. The asterisk (*) indicates a fibrillation score of 0.4. A score>0.4 detected non-responders with moderate sensitivity (64%) and specificity (62%).

The distal CMAP amplitudes at the initial assessment did not differ significantly between responders and non-responders overall (Table 2) and initial distal CMAP amplitudes did not predict treatment outcome. However, non-responders demonstrated progressive decline in CMAP amplitudes on progress nerve conduction studies, suggestive of secondary axonal loss. Using electromyography, fasciculation potentials were common in both groups, but fibrillation potentials, positive sharp waves and complex repetitive discharges were more frequent in non-responders than responders, perhaps indicating more aggressive denervation (i.e. likely ALS). An ROC was constructed to determine the fibrillation score threshold which identified non-responders at an optimal sensitivity and specificity (Figure 2). Using this approach, a fibrillation score of >0.4 was determined to identify non-responders with moderate sensitivity (64%) and specificity (62%).

## Discussion

The present cohort of forty-two patients who presented with an isolated LMN syndrome has identified that the most reliable predictor of a positive IVIg treatment response, and key distinguishing feature from ALS, was the recognition of the typical clinical phenotype of MMN, namely an upper limb, unilateral, and distal onset pattern of weakness. Diagnostic criteria for MMN remain insensitive and would have excluded the majority of responders in this series from a therapeutic trial of therapy. Apart from CB and GM_1_ antibodies, detected in a minority of responders, no neurophysiological or laboratory characteristic reliably distinguished responders from non-responders, with the latter progressing to a diagnosis of ALS. The present study supports the view that patients who present with isolated LMN syndromes should be given an empirical trial of IVIg therapy early in the course of their illness to determine treatment responsiveness.

Due to the lack of demonstrable CB, more than half of the responders in the present series did not satisfy the consensus criteria for the diagnosis of MMN. Nonetheless, responders exhibited a similar clinical phenotype to published cohorts of patients with MMN. [15]–[19] As such, the diagnostic criteria for MMN may be too strict and thus exclude patients with MMN based on an absence of detectable CB. Weakness among responders progressed insidiously, as reflected by symptom durations prior to first assessment, and was predominately upper limb, unilateral, and distal in onset. Responders were younger than non-responders, and as others have observed [17], [20], [21], many responders in the present series had symptoms for several years prior to treatment, often after review by several neurologists. Although not useful in selecting patients for IVIg treatment early in the course of their illness, slow progression of disease over many months or years may allow the distinction of ALS from other conditions. [22] Early intervention in such cases might lead to improved treatment outcomes and potentially reduced long term disability due to secondary axonal loss. If, after early initiation of therapy, patients develop typical features of ALS, such as UMN signs or bulbar involvement, withdrawal of IVIg would be appropriate. Such an approach is becoming the de facto standard of care in many major ALS centres.

The presence of detectable GM_1_ antibodies was highly specific for a positive treatment response to IVIg, but only 12.9% of responders in this series were positive for GM_1_ antibodies. This rate is similar to that documented for patients with LMN syndromes treated with IVIg [20], but lower than in cohorts of MMN. [17], [18] Although the reported sensitivity of GM1 antibodies remains highly variable, GM1 titres have recently been correlated with the severity of weakness in MMN. [23]

Definite or probable CB on standard motor nerve conduction studies was highly specific, but poorly sensitive, for response to IVIg treatment. The relatively low rate of detectable CB in the present series is similar to cohorts of MMN patients. [17], [19] When smaller CMAP amplitude reductions (i.e. ARR) were included in the analysis, the sensitivity improved marginally, but specificity dropped dramatically. Even after including CB, ARR and GM1 antibodies only 45.2% of responders were identified.

Undetected proximal CB among responders in the present series cannot be excluded, as cervical root stimulation was not universally performed. However, cervical root stimulation techniques are technically demanding, and their use in previous studies has yielded variable results. [19], [24] As such, the role of these techniques has not been definitively established. Other techniques, for example utilising transcranial magnetic stimulation may detect proximal CB [25], or conversely, may detect sub-clinical UMN dysfunction in ALS patients. [26], [27] Given the difficulty in detecting proximal CB using neurophysiological techniques, T2 weighted magnetic resonance imaging is recommended by the European Federation Neurological Societies / Peripheral Nerve Society guidelines to establish proximal involvement – such as proximal demyelination or nerve root hypertrophy – in MMN or other immune mediated neuropathies. [28], [29]

In summary, the presence of CB or GM_1_ antibodies are specific but insensitive predictors of response to IVIg in patients that present with isolated LMN syndromes that do not meet diagnostic criteria for degenerative motor neuron diseases or inflammatory motor neuropathy. Responders may have slowly progressive symptoms for many years prior to starting treatment, that may detrimentally affect the therapeutic outcome. Given that the present cohort may be considered relatively small, a larger randomised placebo control trial would be required to definitively establish the role of IVIg in patients with pure LMN syndromes that do not fulfil diagnostic criteria. Nonetheless, the present study supports the view that patients with pure LMN syndromes should be given an early therapeutic trial of IVIg, even in the absence of GM_1_ antibodies and CB.
